# Metagenomic and Metatranscriptomic Analyses Revealed Uncultured Bacteroidales Populations as the Dominant Proteolytic Amino Acid Degraders in Anaerobic Digesters

**DOI:** 10.3389/fmicb.2020.593006

**Published:** 2020-10-30

**Authors:** Ran Mei, Masaru K. Nobu, Takashi Narihiro, Wen-Tso Liu

**Affiliations:** ^1^Department of Civil and Environmental Engineering, University of Illinois at Urbana-Champaign, Urbana, IL, United States; ^2^Bioproduction Research Institute, National Institute of Advanced Industrial Science and Technology (AIST), Tsukuba, Japan

**Keywords:** metagenomics, uncultured, proteolytic, amino acid, anaerobic digester

## Abstract

Current understanding of amino acid (AA) degraders in anaerobic digesters is mainly based on cultured species, whereas microorganisms that play important roles in a complex microbial community remain poorly characterized. This study investigated short-term enrichments degrading single AAs using metagenomics and metatranscriptomics. Metagenomic analysis revealed that populations related to cultured AA degraders had an abundance <2.5% of the sequences. In contrast, metagenomic-assembled bins related to uncultured *Bacteroidales* collectively accounted for >35% of the sequences. Phylogenetic analyses suggested that these *Bacteroidales* populations represented a yet-to-be characterized family lineage, i.e., *Bacteroidetes* vadinHA17. The bins possessed the genetic capacity related to protein degradation, including surface adhesion (3–7 genes), secreted peptidase (52–77 genes), and polypeptide-specific transporters (2–5 genes). Furthermore, metatranscriptomics revealed that these *Bacteroidales* populations expressed the complete metabolic pathways for degrading 16 to 17 types of AAs in enrichments fed with respective substrates. These characteristics were distinct from cultured AA degraders including *Acidaminobacter* and *Peptoclostridium*, suggesting the uncultured *Bacteroidales* were the major protein-hydrolyzing and AA-degrading populations. These uncultured *Bacteroidales* were further found to be dominant and active in full-scale anaerobic digesters, indicating their important ecological roles in the native habitats. “*Candidatus* Aminobacteroidaceae” was proposed to represent the previously uncharted family *Bacteroidetes* vadinHA17.

## Introduction

Anaerobic digestion (AD) is a biological process where complex polymers are converted to small molecules and mineralized to methane and carbon dioxide. Protein is one of the polymers and accounts for a significant portion (25.4%–37.9% of the volatile solids content) of organic feed to anaerobic digesters (McInerney, 1988). It is also abundant in wastewater produced from food industries that process whey, cheese, and fish (Tang et al., 2005). Protein degradation can be the rate-limiting step in AD, and has been shown as a slower process than carbohydrates degradation (Gujer and Zehnder, 1983; Yang et al., 2015). Protein is subsequently hydrolyzed to amino acids (AAs), which are the key groups of intermediates in AD metabolism. Despite the importance of AAs in anaerobic environments, few studies explicitly identify microorganisms that can degrade AAs especially in a complex microbial community like AD (Tang et al., 2005). Furthermore, AA-degrading microorganisms were primarily studied in the 1980s based on clostridia pure cultures (McInerney, 1988). Considering most anaerobic microorganisms have not been cultured, important AA degraders in AD remain to be uncovered.

Our recent study characterized microbial communities that were inoculated with AD sludge and enriched with individual AAs (Mei et al., 2020). During continuous transfer spanning 18 months, analyses of 16S rRNA and rRNA gene revealed a shift in predominant populations from uncultured members of the order *Bacteroidales* in the early enrichment to cultured AA fermenters including *Acidaminobacter* and *Peptoclostridium* at the end of enrichment. This observation implied that although continuous enrichment is the classic strategy to characterize novel microorganisms, it may fail to capture species that are functionally important in the native habitats due to the bias associated with artificial cultivation conditions (Kamagata, 2015). The predominance of uncultured *Bacteroidales* populations (>20% 16S rRNA abundance) in the short-term enrichment communities further indicated their important roles involved in AA degradation.

The order *Bacteroidales* is one of the most prevalent in AD, and its abundance can reach as high as 15% in a community (Mei et al., 2016, 2018; Narihiro et al., 2018; Zealand et al., 2018; Nobu et al., 2020). It is also widely distributed in different non-AD habitats (Gupta, 2004) such as animal intestinal microflora (Dick et al., 2005) and marine bacterioplankton (Fernández-Gomez et al., 2013). Many studies (Narihiro et al., 2015; Kim et al., 2016; Ormerod et al., 2016; Ramayo-Caldas et al., 2016; Vanwonterghem et al., 2016; Reichardt et al., 2017; Lagkouvardos et al., 2019) have suggested populations related to *Bacteroidales* as saccharolytic because carbohydrate degradation is a common feature among most non-marine isolated species of this order (Rosenberg et al., 2014). However, there are still more than 20 uncultured family-level lineages in this order based on the most recent SILVA database (release 138) (Yilmaz et al., 2013), including *Bacteroidetes* vadinHA17, *Bacteroidetes* BD2-2, and *Bacteroidales* RF16, suggesting the metabolic diversity of this order is not fully uncovered. Some of those uncharacterized *Bacteroidales* are likely capable of protein and amino acids degradation given their high abundances observed in anaerobic reactors treating protein-rich substrates such as bovine serum albumin (Tang et al., 2005) and casein (Ley et al., 2006). In addition, a few *Bacteroidales* isolates have been reported to be capable of non-saccharolytic metabolisms [e.g., *Proteiniphilum saccharofermentans* (Tomazetto et al., 2018), *Williamwhitmania taraxaci* (Pikuta et al., 2017), and *Salinivirga cyanobacteriivorans* (Ben Hania et al., 2017)]. Therefore, further investigation is clearly warranted to expand our understanding about the diverse roles that uncultured *Bacteroidales* could play, including protein and amino acids degradation.

The present study conducted metagenomic and metatranscriptomic analyses to decipher the roles of the uncultured *Bacteroidales* that were abundant in the short-term enrichment communities we previously established (Mei et al., 2020). The combination of short-term enrichment and meta-omics analyses can exert sufficient selective pressure while avoiding enrichment bias. Functions related to protein and AA degradation were assessed in detail based on the recovered genomes and gene expression profile of the *Bacteroidales* populations. Their ecological prevalence in full-scale ADs and sediment environments was also evaluated.

## Materials and Methods

### Enrichment Cultures and Sequencing

Enrichment cultures were described in our previous publication (Mei et al., 2020). In brief, anaerobic digester sludge taken from a municipal wastewater treatment plant (Urbana, IL, United States) was used as inoculum (2 mL sludge into 80 mL media). Twenty individual amino acids were used individually as single substrates (5 mM final concentration). The cultures were incubated under 35°C in dark without strong mixing to mimic the anaerobic digester environment. When the substrates were used up, as determined by ceased methane production, and prior to further culture transfer, 14 of the 20 enrichments were selected for metagenomic sequencing and 19 for metatranscriptomic sequencing (with duplicates, Supplementary Table 1). Sequencing was performed at the Joint Genomic Institute (JGI) in US Department of Energy using the Illumina HiSeq-2500 1TB platform. The raw sequences are available at the JGI Genome Portal (Supplementary Table 1). JGI also performed sequencing filtering and assembling. For metagenomic sequences, BBDuk v38.08 was used to remove contaminants and trim reads that contained adapter sequence and had low quality^1^. Reads mapped to masked human, cat, dog and mouse references and other common microbial contaminants were also removed. Filtered reads were further corrected using BFC vr181 (Li, 2015) and assembled using SPAdes assembler v3.11.1 (Nurk et al., 2017). For metatranscriptomic sequences, quality filtering was performed in the same way as metagenomic sequences with additional removal rRNA reads.

### Metagenomic Binning

Two binning softwares, MetaBAT v2.12.1 (Kang et al., 2015) and MaxBin v2.2.5 (Wu et al., 2015), were applied to the metagenomic assembly and generated two independent sets of bins from each sample. Binning_refiner v1.2 (Song and Thomas, 2017) was used to compare and merge the results of MetaBAT and MaxBin. The resulting bins were further improved using RefineM v0.0.24 (Parks et al., 2017). The quality, i.e., completeness and contamination, of the final bins was assessed using CheckM v1.0.12 (Parks et al., 2015). Bins with >80% completeness and <5% contamination, were selected for downstream analysis. Phylophlan v0.99 (Segata et al., 2013) was used to identify and align marker proteins from bins. The alignment was further used to construct a phylogenomic tree using maximum likelihood method with bootstrap value of 100 in MEGA X (Kumar et al., 2018). Average amino acids identity (AAI) between bins was calculated using CompareM v0.0.23 (Parks et al., 2017). Based on the results of phylogenomics and AAI, closely related bins from different samples were grouped using Spine (Ozer et al., 2014) with only core genome content being kept. In total, 56 final bins were obtained including pangenomes. The taxonomy of the final bins was further assessed using GTDB-TK v1.0.2 (Chaumeil et al., 2019). Quality-filtered metagenomic and metatranscriptomic reads were mapped to the 56 final bins using BBmap v38.46 to estimate the representativeness of the genomes. The metagenomic abundance of the genomes in the community was calculated as described previously (Woodcroft et al., 2018). Briefly, quality-filtered metagenomic reads from each sample were mapped to the genomes using BamM v1.7.3 (Parks et al., 2017). Low-quality mappings were removed, and the length-weighted coverage of each contig was averaged to calculate the coverage of the genomes in the community. The relative abundance of each genome in each sample was calculated as its coverage divided by the total coverage of all the genomes. The draft genomes were deposited in NCBI GenBank under the accession WXFB00000000-WXFH00000000. 16S rRNA genes found in the genomes were deposited under the accession MK990229–MK990231.

### Comparative Genomics

Five *Bacteroidales* bins and two bins related to *Peptoclostridium* and *Acidaminobacter* were analyzed in detail. Initial annotation was performed with Prokka v1.13.3 (Seemann, 2014). The 16S rRNA gene sequences obtained from *Bacteroidales* bin 2, 3, and 4 were analyzed in ARB (Ludwig et al., 2004) using maximum likelihood method RAxML with 100 time bootstrapping. Phylogenomic analysis was performed using Phylophlan v0.99 and MEGA X. The annotated genomes were then checked and corrected manually by searching for similar proteins (>50% similarity and >50% coverage) in the UniProt Knowledgebase^2^ using BLASTP v2.10.0. Peptidase, lipase, transporter, hydrogenase were identified by BLASTP v2.10.0 search using the MEROPS database release 12.1 (Rawlings et al., 2017), Lipase Engineering Database (Widmann et al., 2010), Transporter Classification Database (Saier et al., 2015), HydDB (Søndergaard et al., 2016), with >50% amino acids similarity and >50% coverage. Carbohydrate-active enzyme and signal peptide were annotated using dbCAN2 (Zhang et al., 2018) and SignalP 5.0 (Armenteros et al., 2019). Genes related to surface adhesion were found using the Pfam search as described previously (Fernández-Gomez et al., 2013). SusC/RagA-like protein sequences described previously (Ben Hania et al., 2017) were obtained from GenBank or Uniprot. Alignment of the protein sequences was performed using ClustalW, and phylogenetic analysis was performed using maximum likelihood algorithm with 100 bootstraps implemented in MEGA X. BBmap v38.46 was used to calculate the gene expression levels as reads per kilobase transcript per million reads (RPKM). Public metagenomic and metatranscriptomic sequences obtained from full-scale anaerobic digesters (Nobu et al., 2020) or sediments (Urich et al., 2014; Dyksma et al., 2016; Voorhies et al., 2016; Chu et al., 2018; Orsi et al., 2018) were mapped to the bins to estimate prevalence of the studied populations.

## Results and Discussion

### AA-Degrading Community Structure Based on Metagenomics

Among 20 short-term enrichment cultures degrading individual AAs established in our previous study (Mei et al., 2020), 14 were further selected for metagenomic sequencing (Supplementary Table 1). The remaining cultures were not selected because they had very similar 16S rRNA gene-based community structures (i.e., cultures fed with cysteine, serine, aspartic acid, glutamine, and proline) or had no growth (i.e., cultures fed with tyrosine). From the 14 metagenomic datasets, a total of 796 metagenomics-assembled bins were obtained and 281 bins with >80% completeness and <5% contamination were selected for downstream analysis (Supplementary Figure 1). After merging bins closely clustered with each other based on phylogenomic analysis, 56 final bins were obtained. These 56 final bins showed good representativeness of the populations in the enrichment cultures as majority of the metagenomic (67.4% on average) and metatranscriptomic reads (86.2% on average) in each culture could be mapped to them (Supplementary Figure 2).

**FIGURE 1:**
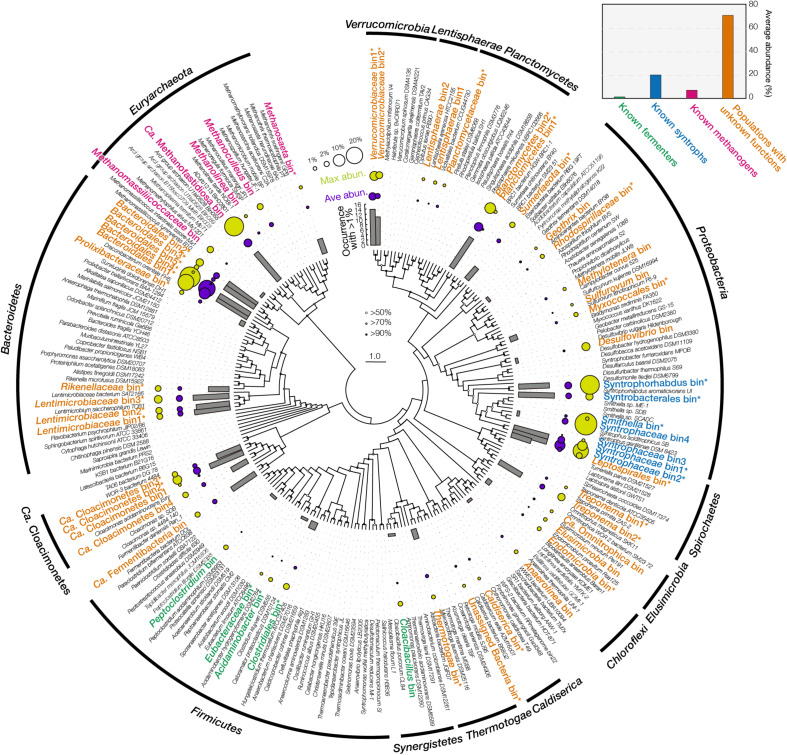
Fifty-six final bins assembled from metagenome. The phylogenetic tree was built using maximum likelihood method with 100 bootstraps. Bootstrap values are denoted using bubbles on the node. Bars in the inner circle denote the occurrence of each bin with an abundance >1% among the 14 metagenomic datasets. Bubbles in the middle circle denote the average metagenomic abundance. Bubbles in the outer circle denote the maximum metagenomic abundance. Bins’ name is colored according to their potential functions. Asterisk after the bins’ name indicates this final bin was merged from multiple bins from different samples clustered together in this tree. Small panel at the top-right corner shows the average abundance of four functional groups.

Figure 1 showed the metagenomic abundances of those 56 bins. Among them, five bins were related to known AA fermenters, such as *Peptoclostridium acidaminophilum* and *Acidaminobacter hydrogenoformans*. They had low abundance (total abundance of 1.2%) and low occurrence (detected in < 3 cultures with > 1% abundance), suggesting the marginal roles of these populations in the short-term enrichment. Seven bins were related to syntrophic acids degraders (total abundance of 20%), including *Syntrophaceae* (7.7% on average), *Syntrophorhabdus* (3.5%), and *Syntrophobacter* (2.4%). These syntrophs could degrade diverse intermediates that were produced from AA fermentation and biomass degradation (Nobu et al., 2015). Specifically, the bins related to *Syntrophaceae* and *Syntrophorhabdus* had a high abundance (maximum abundance > 15%) in cultures fed with leucine, isoleucine, valine, tryptophan, or phenylalanine (Supplementary Figure 3), suggesting they played important roles in degrading intermediates generated from specific AA fermentation, such as branch-chain fatty acids, heterocyclic compounds, and phenolic compounds. For methanogens, five bins were detected (total abundance of 7%), including those related to *Methanosaeta* (0.5% on average), *Methanoculleus* (1.3%), and *Ca.* Methanofastidiosa (2.7%). They represented different types methanogenic pathways, i.e., aceticlastic, hydrogenotrophic, and methyl-reducing. Specifically, bins related to *Ca.* Methanofastidiosa (26.6%) and *Methanomassiliicoccaceae* (3.1%) were abundant in the culture fed with methionine, consistent with previous reports that they could reduce methylated compounds and produce methane (Dridi et al., 2012; Nobu et al., 2016). Last, populations with unknown functions were consistently observed with high abundance (total abundance of 72%) in all the 14 enrichments, including five bins related to the order *Bacteroidales.* Among the five *Bacteroidales* bins, bin1, bin2, and bin4 were more abundant (9.1%, 2.6%, and 20.2%, respectively) than bin3 (0.8%) and bin5 (0.2%).

### Phylogenetic Analysis of *Bacteroidales* Bins

**FIGURE 2:**
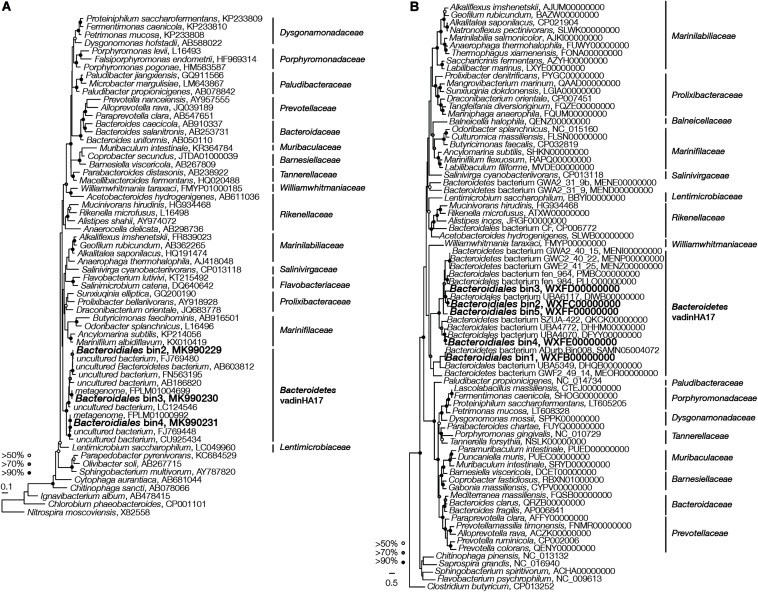
Phylogenetic analysis of *Bacteroidales* bins based on **(A)** 16S rRNA gene sequences and **(B)** universally conserved protein sequences.

Phylogenetic affiliations of the *Bacteroidales* bins were examined by first analyzing the nearly full-length 16S rRNA gene sequences found in three of the five *Bacteroidales* bins (bins 2, 3, and 4). We did not find 16S rRNA genes in bins 1 and 5 after several trials. These *Bacteroidales* bins were distinct from all known *Bacteroidales* families containing cultured representatives (Figure 2A), but were affiliated with an uncultured family-level lineage *Bacteroidetes* vadinHA17. The sequence similarity between the three 16S rRNA gene sequences and representative sequences of other *Bacteroidales* families ranged from 79.2% to 88.6% (Supplementary Table 2), suggesting that vadinHA17 represented a novel family based on the sequence similarity threshold of 86.5% for family and 94.5% for genus (Yarza et al., 2014). Analysis based on universally conserved protein sequences further supported that the five *Bacteroidales* bins formed a cluster that was distinct from all cultured *Bacteroidales* families (Figure 2B). The average amino acid identity (AAI) of the five *Bacteroidales* bins against representative genomes of cultured families ranged from 49.7% to 57.6% (Supplementary Table 2), indicating that they were not associated with existing cultured families based on a cutoff of ∼60% (Konstantinidis and Tiedje, 2005).

### Protein Degradation by the *Bacteroidales* Species

**FIGURE 3:**
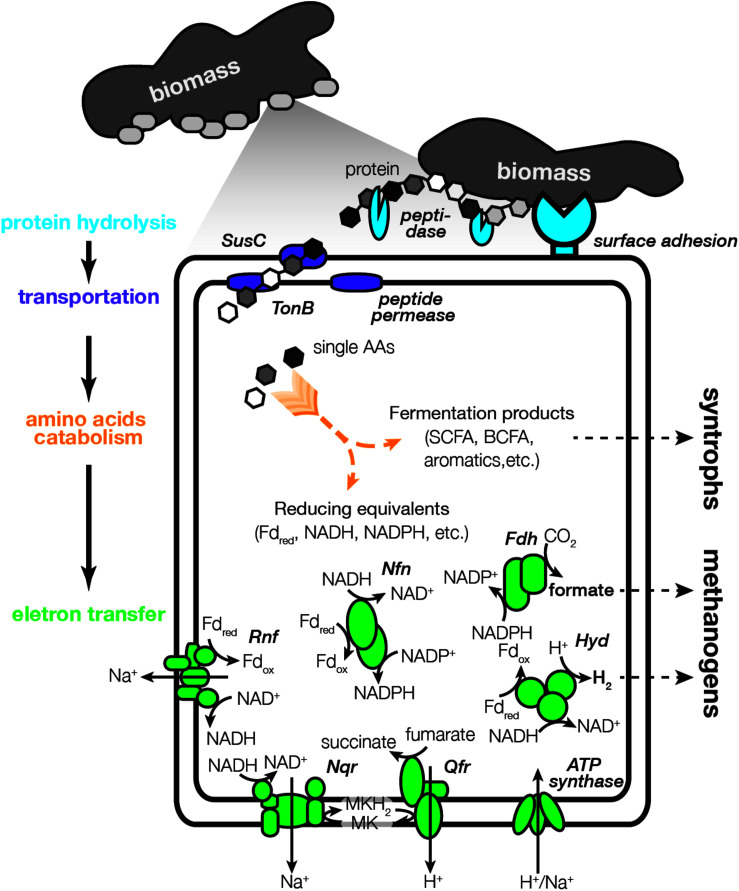
Illustration of the proteolytic amino acids degrading lifestyle of the *Bacteroidales* populations.

Figure 3 shows the general lifestyle of protein degradation, AA metabolism and energy conservation based on the annotation of *Bacteroidales* bins 1, 2, and 4 that were abundant in the cultures (abundance >2%). In addition, two bins that were closely related to cultured AA degraders, i.e., *Acidaminobacter* (Stams and Hansen, 1984) and *Peptoclostridium* (Zindel et al., 1988), were analyzed and used us references. Domains involved in surface adhesion to biomass were first examined (Fernández-Gomez et al., 2013). The *Bacteroidales* bins were observed to encode more adhesion genes than the cultured AA degraders (Supplementary Table 3). Bins 1, 2, and 4 encoded 3, 6, and 7 adhesion genes, including the Bac_surface_Ag family (PF01103) related to outer membrane surface antigen, the F5_F8_Type_C family (PF00754) related to cell adhesion protein discoidin and the HYR family (PF02494) related to cell adhesion (Baumgartner et al., 1998; Callebaut et al., 2000; Feng et al., 2012). Particularly, genes related to the HYR family had the highest expression in the three bins (2.3-4.4 times of the bins’ median activity), suggesting the critical role of the cell adhesion function. In contrast, the *Acidaminobacter* bin and *Peptoclostridium* bin did not encode genes related to those families. The *Acidaminobacter* bin only encoded one gene related to the fasciclin family (PF02496), an ancient cell adhesion domain (Huber and Sumper, 1994). These observations suggested that the *Bacteroidales* populations had distinct surface adhesion properties from cultured AA degraders.

After biomass adhesion, we analyzed extracellular peptidases that can hydrolyze the protein fractions in the biomass (Figure 3). Bins 1, 2, and 4 encoded 172, 109, and 100 peptidase genes, respectively (Supplementary Table 4). Metallo (M) and serine (S) peptidases were the most abundant types (>40 genes), followed by cysteine (C) peptidases (>15 genes) and small numbers of other types (<5 genes). These genes included known extracellular peptidases such as papain (C01A), thermitase (S08A), and tricorn core protease (S41B). To specifically identify peptidases that were secreted out of the membrane and involved in protein hydrolysis, putative signal sequence motifs that translocate peptidases toward the secretory system were examined (Nguyen et al., 2019). Bins 1, 2, and 4 encoded 72, 55, and 68 secreted peptidase genes, respectively, higher than the *Acidaminobacter* bin (12 genes) and *Peptoclostridium* bin (8 genes). Among secreted peptidases that were encoded, bins 1, 2, and 4 highly expressed 38, 16, and 30 genes, respectively (> the bins median activity) (Supplementary Figure 4), significantly higher than the number expressed by the *Acidaminobacter* bin (2.4 genes on average) and *Peptoclostridium* bin (0.3 genes on average). The *Bacteroidales* bins also encoded genes for secreted carbohydrates and lipids hydrolysis. But these genes had lower expression level compared with secreted peptidases, suggesting the *Bacteroidales* were specialized in hydrolyzing protein but not polysaccharides or lipids in the short-term enrichments. Their activities in degrading other macromolecule substrates in different environments remain further investigation.

After protein hydrolysis, the *Bacteroidales* species were observed with the capacity of transporting protein hydrolysis products using the TonB-dependent receptor SusC protein (Figure 3). While SusC protein is usually considered to be related to polysaccharide uptake and transport (Reeves et al., 1997), recent evidences also revealed its role in taking up non-polysaccharide substrates such as peptides (Ben Hania et al., 2017) and other degradation products of protein (Nagano et al., 2007). The types of substrates that SusC protein utilized can be also inferred from its sequence similarity (Ben Hania et al., 2017). Bins 1, 2, and 4 encoded 3 to 8 susC genes, and more than half were located in a cluster specialized for polypeptide transport based on the phylogenetic analysis of SusC protein sequences found in diverse *Bacteroidetes* genomes (Supplementary Figure 5). This polypeptide-specific cluster also included most *Bacteroidales* species known to utilize proteinaceous substrates, including *Williamwhitmania taraxaci* (Pikuta et al., 2017), *Porphyromonas gingivalis* (Nagano et al., 2007), *Acetobacteroides hydrogenigenes* (Su et al., 2014), and *Proteiniphilum saccharofermentans* (Tomazetto et al., 2018). We also found TonB proteins and oligopeptide permeases in *Bacteroidales* bins, which could facilitate the cross-membrane transport of protein hydrolysis products. In contrast, no susC gene was identified in the *Acidaminobacter* bin and *Peptoclostridium* bin. Overall, the characterization of biomass adhesion, secreted peptidase, and cross-membrane transporter strongly suggested that the *Bacteroidales* populations had a proteolytic lifestyle, which was not observed with *Acidaminobacter* and *Peptoclostridium* that degrade soluble AA monomers. They were dominant in the short-term enrichment because there were plenty of insoluble macromolecules in the inoculum biomass (2.5 ml anaerobic digester sludge). These observations were consistent with our previous results that the *Bacteroidales* populations were competed out after long-term enrichment where biomass was significantly diluted after a series of transfer (Mei et al., 2020).

### Amino Acids Metabolism by the *Bacteroidales* Species

The *Bacteroidales* species were further observed to possess the capacity to metabolize individual AAs that were available in the cell (Figure 3). Catabolic pathways of each AA were manually identified, and one pathway was defined as complete only when all the genes of this pathway were found in the bins. For example, bins 1, 2, and 4 were identified to be able to degrade alanine because they encoded the gene for alanine dehydrogenase, a key enzyme that converts alanine to pyruvate (Supplementary Table 5 and Supplementary Figure 6). For AAs that require multi-step pathways such as glycine, bins 1, 2, and 4 could degrade glycine to pyruvate because all the six genes of the glycine decarboxylating pathway were identified in each bin. In total, bins 1, 2, and 4 were observed to encode the complete pathways for degrading 16, 17, and 17 types of AAs, respectively (Supplementary Table 6). Such genetic capacity was comparable to the *Acidaminobacter* bin and *Peptoclostridium* bin, which possessed pathways to degrade 13 and 16 types of AAs, respectively.

**FIGURE 4:**
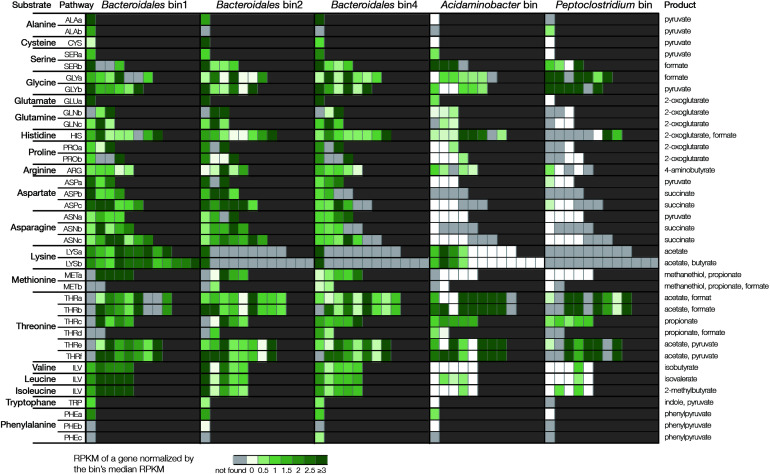
Gene expression of amino acids degradation pathways in the respective cultures. Each substrate can have multiple pathways (details of the pathways are listed in Supplementary Table 5). Genes in each pathway are represented by individual cells. Light gray color indicates the gene is not found in the bins. Color scale from white to green indicates the level of expression based on normalized RPKM value.

The expression of these pathways was further examined in the enrichments fed with respective substrate based on metatranscriptomics (Figure 4). For example, in the enrichment fed with alanine, all three *Bacteroidales* bins expressed alanine dehydrogenase whereas the *Acidaminobacter* bin and the *Peptoclostridium* bin did not express the gene. In the enrichment fed with glycine, bin1 and bin4 expressed all the six genes of the glycine decarboxylating pathway. In contrast, there were at least one gene not expressed by bin2, the *Acidaminobacter* bin, and the *Peptoclostridium* bin. In summary, bins 1, 2, and 4 expressed the complete degradation pathways of 16, 11, and 17 types of AAs (Supplementary Table 6). These gene expression results were supported by the abundance profile that bin1 and bin4 were more abundant than bin2 (9.1% and 20.2% vs. 2.6%). As a comparison, the bins related to *Acidaminobacter* and *Peptoclostridium* only expressed the pathways of five and two types of AAs, respectively, which was in line with their low abundances (<0.5%). These results indicated that although *Acidaminobacter* and *Peptoclostridium* were well-known AA degraders, the *Bacteroidales* populations were the actual active degraders in the short-term enrichment culture.

### Electron Transfer by the *Bacteroidales* Species

Figure 3 further shows the mechanisms that the *Bacteroidales* populations used to transfer electrons and conserve energy. During AA metabolism, electrons are generated and carried by reducing equivalents such as reduced ferredoxin, NADH, and NADPH. The *Bacteroidales* bins possessed diverse approaches to reoxidize these electron carriers for sustainable fermentation and simultaneous energy conservation (Supplementary Table 7). In the cytoplasm, genes related to hydrogen production (Hyd) and formate production (Fdh) were consistently expressed, which allowed the *Bacteroidales* bins to use proton or CO_2_ as the sink for electron disposal. The production of hydrogen and formate was further supported by the consistent detection of hydrogen- and formate-utilizing methanogens including *Methanolinea, Ca.* Methanofastidiosa, and *Methanoculleus* in the enrichments. In terms of membrane-bound enzymes, the *Bacteroidales* bins contained Rnf-type ion-translocating NADH:ferredoxin oxidoreductase complex that can leverage the low-potential reduced ferredoxin to generate a sodium gradient for ATP synthesis. Similarly, the quinol:fumarate oxidoreductase can couple with the NADH:quinone oxidoreductase to translocate proton and sodium ion across the membrane, which can be further used for energy conservation. These electron transfer and energy conservation mechanisms suggested that the *Bacteroidales* populations had an AA-fermentation lifestyle in the enrichment cultures.

### Micro-Heterogeneity Among *Bacteroidales* Species

Differences in AA metabolism were further observed between individual *Bacteroidales* bins. Bin2 and bin4 were more similar compared to bin1. Among the tested AAs, bin2 and bin4 shared most degradation pathways. Also, they both missed alanine transaminase in alanine degradation, glutamate synthase large chain in glutamine degradation, 1-pyrroline-5-carboxylate dehydrogenase in proline degradation, and eight genes in lysine degradation (Supplementary Table 5). In contrast, bin1 encoded the genes that were missing in bin2 and bin4, including alanine transaminase, 1-pyrroline-5-carboxylate dehydrogenase, and the entire lysine degradation pathway. On the other hand, bin1 missed genes that were detected in both bin2 and bin4, including methenyltetrahydrofolate cyclohydrolase for histidine degradation, formate C-acetyltransferase for methionine degradation, and the entire threonine degradation capacity due to the lack of L-threonine aldolase, threonine 3-dehydrogenase, and L-threonine ammonia-lyase. In addition, bin1 was observed in a sister cluster with bin2 and bin4 in the genome tree (Figure 2), implying heterogeneity at sub-family level within the clade vadinHA17. However, due to the lack of 16S rRNA gene in bin1, we were not able to accurately determine whether bin1 represent a different species or genus. The difference in phylogeny was consistent with other physiological differences. For example, bin1 expressed less glycoside hydrolases (21 genes) and lipases (3 genes) than bin2 (57 and 12 genes) and bin4 (41 and 12 genes) (Supplementary Figure 4). Bin1 also encoded one susC gene for α-glucan transportation, whereas bin2 and bin4 encoded susC genes specific for β-glucan transportation (Supplementary Figure 5). Overall, based on the phylogenetic affiliation and metabolic features of the *Bacteroidales* bins, we propose the provisional name “*Candidatus* Aminobacteroidaceae” fam. nov. for the lineage previous known as *Bacteroidetes* vadinHA17 and “*Candidatus* Aminobacteroides proteolyticus” gen. nov. sp. nov. for *Bacteroidales* bin4, the most abundant bin found in the enrichment culture.

### Ecological Prevalence of Uncultured *Bacteroidales*

**FIGURE 5:**
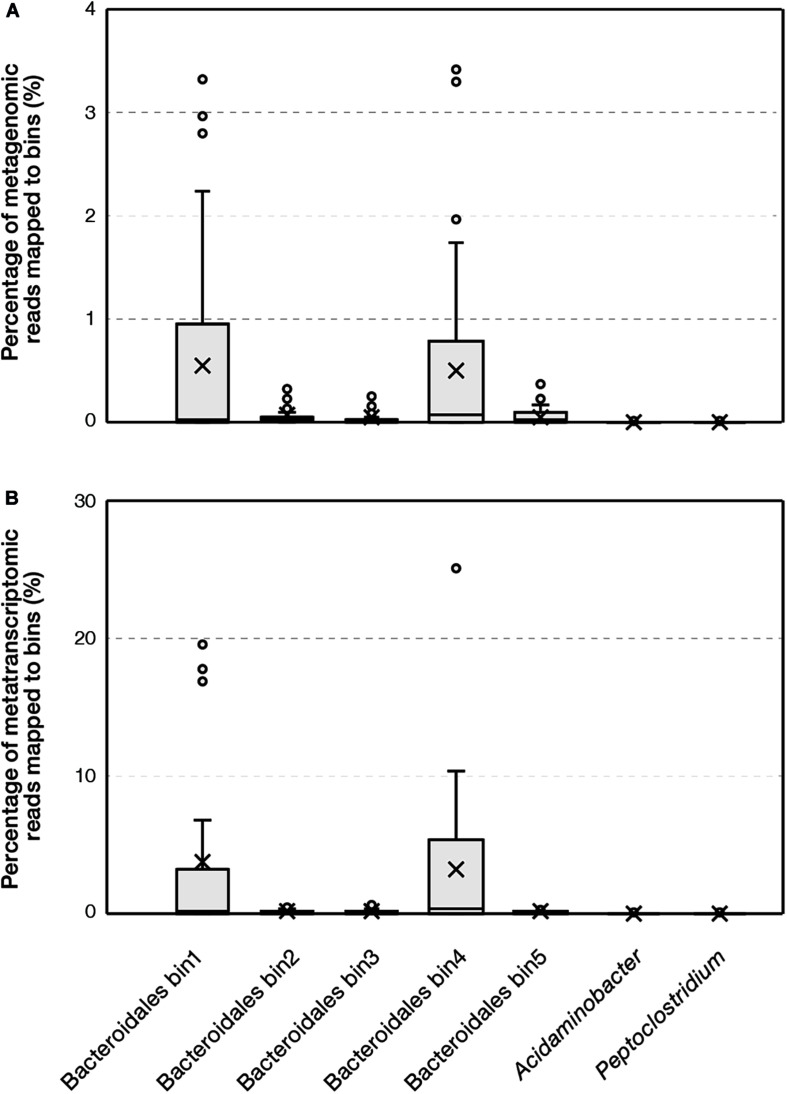
Percentage of reads that were mapped to bins based on **(A)** metagenomic and **(B)** metatranscriptomic datasets obtained from full-scale anaerobic digesters.

We further evaluated the environmental prevalence of the family *Ca.* Aminobacteroidaceae (i.e., *Bacteroidetes* vadinHA17) represented by the *Bacteroidales* bins recovered in this study. This family contained 333 full-length 16S rRNA gene sequences in the most recent release of SILVA NR database. The majority of sequences were obtained from sediments (150 sequences) and anaerobic bioreactors (140 sequences), where proteinaceous substrates are abundant. The abundance of *Ca.* Aminobacteroidaceae populations was further reported to reach over 15% of the microbial community in up-flow anaerobic sludge bed reactors (Chen et al., 2017; Wang et al., 2018), suggesting their important roles in anaerobic environments. Furthermore, we mapped metagenomic and metatranscriptomic sequences obtained from full-scale ADs and sediments to the *Ca.* Aminobacteroidaceae bins. Among 55 metagenomic datasets retrieved from full-scale ADs, the bins were consistently detected (Figure 5). Bin1 and bin4 were abundant, accounting for up to 3.5% of metagenomics sequences. Among 24 metatranscriptomic datasets retrieved from full-scale ADs, the maximum abundances of bin1 and bin4 were >25%. In comparison, the bins related to *Acidaminobacter* and *Peptoclostridium* only accounted for <0.01% of metagenomic or metatranscriptomic sequences. Overall, the abundance of *Ca.* Aminobacteroidaceae bins based on metagenomic and metatranscriptomic sequences suggested they were the dominant populations that performed proteolytic AA degradation in full-scale AD ecosystems. In contrast, among 26 metagenomic and 13 metatranscriptomic datasets retrieved from marine and lake sediments, the five *Bacteroidales* bins were not detected in most samples and the maximum abundance was < 0.03%. The results suggested that these *Ca.* Aminobacteroidaceae populations were unique to AD environments whereas the niche of protein and AA degradation in sediments could be occupied by other microorganisms.

## Data Availability Statement

The datasets presented in this study can be found in online repositories. The names of the repository/repositories and accession number(s) can be found in the article/ Supplementary Material.

## Author Contributions

RM designed the study, conducted the experiments, analyzed the data, and wrote the manuscript. MN, TN, and W-TL designed the study, collaboratively interpreted the results, and revised the manuscript. All authors contributed to the article and approved the submitted version.

## Conflict of Interest

The authors declare that the research was conducted in the absence of any commercial or financial relationships that could be construed as a potential conflict of interest.

## Footnotes

1 https://sourceforge.net/projects/bbmap/

2 Uniref100, https://www.uniprot.org/.
