# Supplementary material for: Metagenomic and Metatranscriptomic Analyses Revealed Uncultured Bacteroidales Populations as the Dominant Proteolytic Amino Acid Degraders in Anaerobic Digesters
Source: Front Microbiol. 2020 Oct 30;11:593006. doi: 10.3389/fmicb.2020.593006 (PMC7661554; doi:10.3389/fmicb.2020.593006)
Supplement: Supplementary file 1 [file Table_1.DOCX]

Supplementary Material

# Supplementary Figures and Tables

## Supplementary Figures


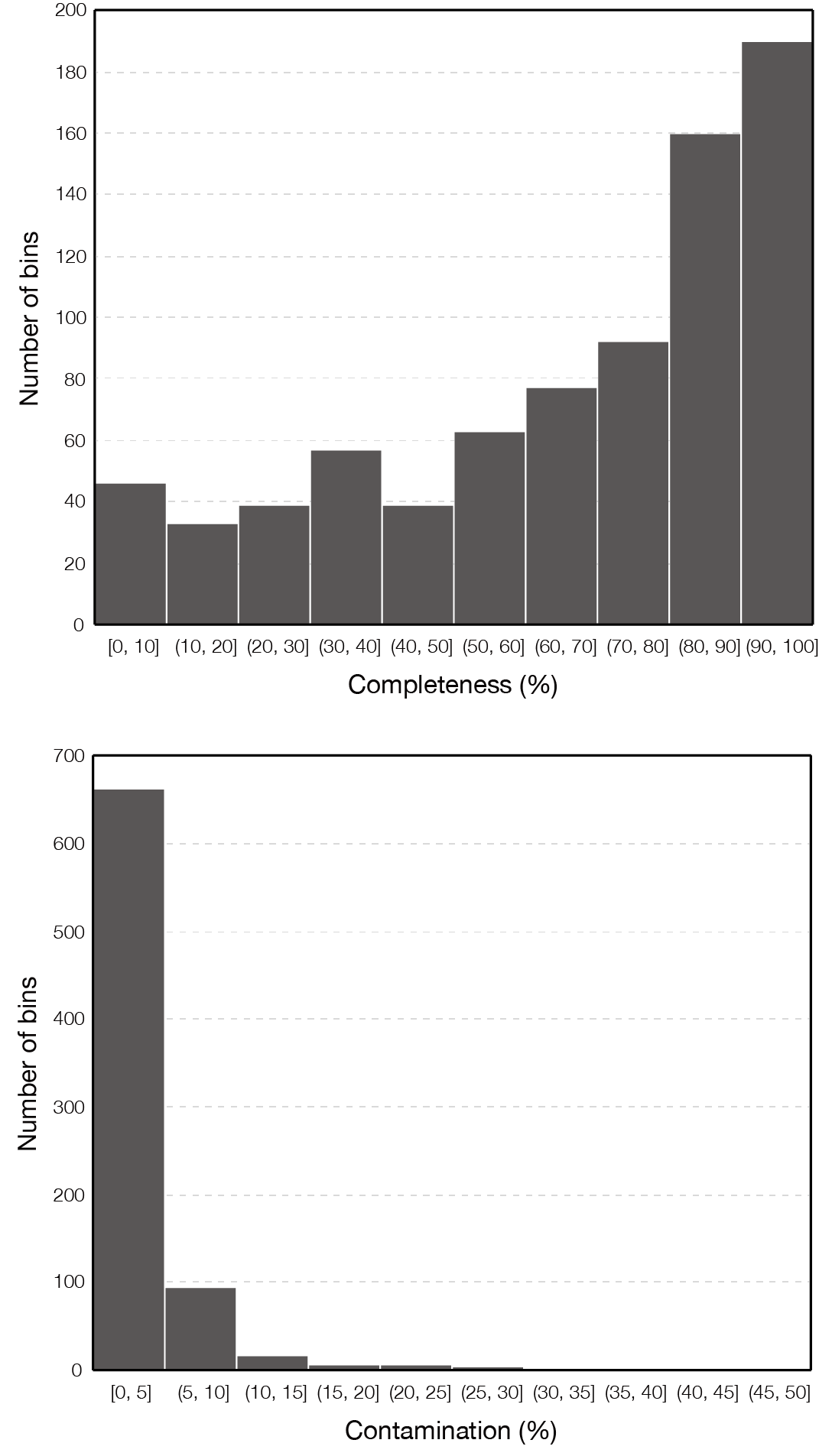


**Supplementary Figure 1.** Histograms of completeness and contamination of the 796 metagenomic-assembled bins.


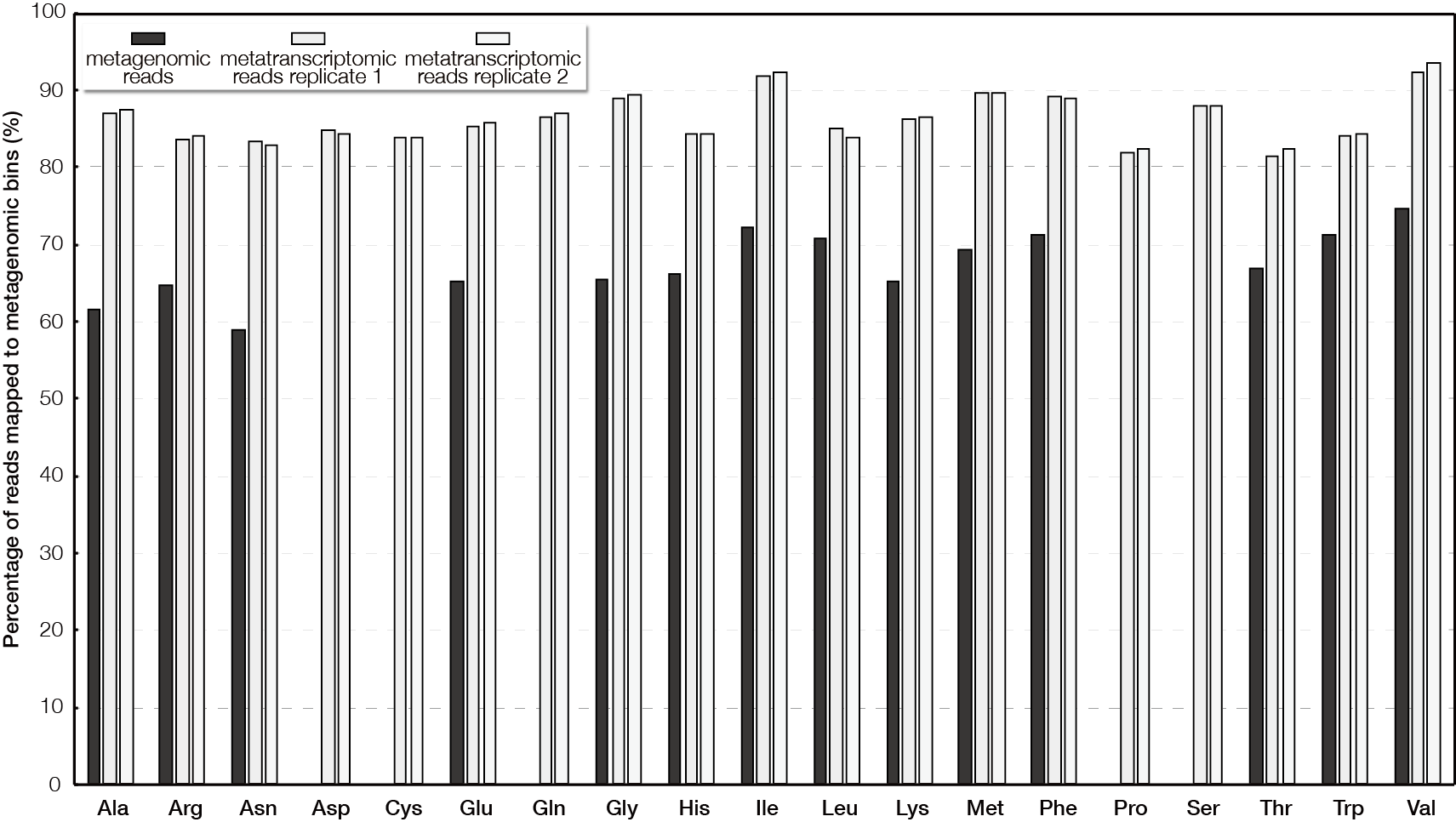


**Supplementary Figure 2.** Percentage of metagenomic and metatranscriptomic reads mapped to the 56 final bins.


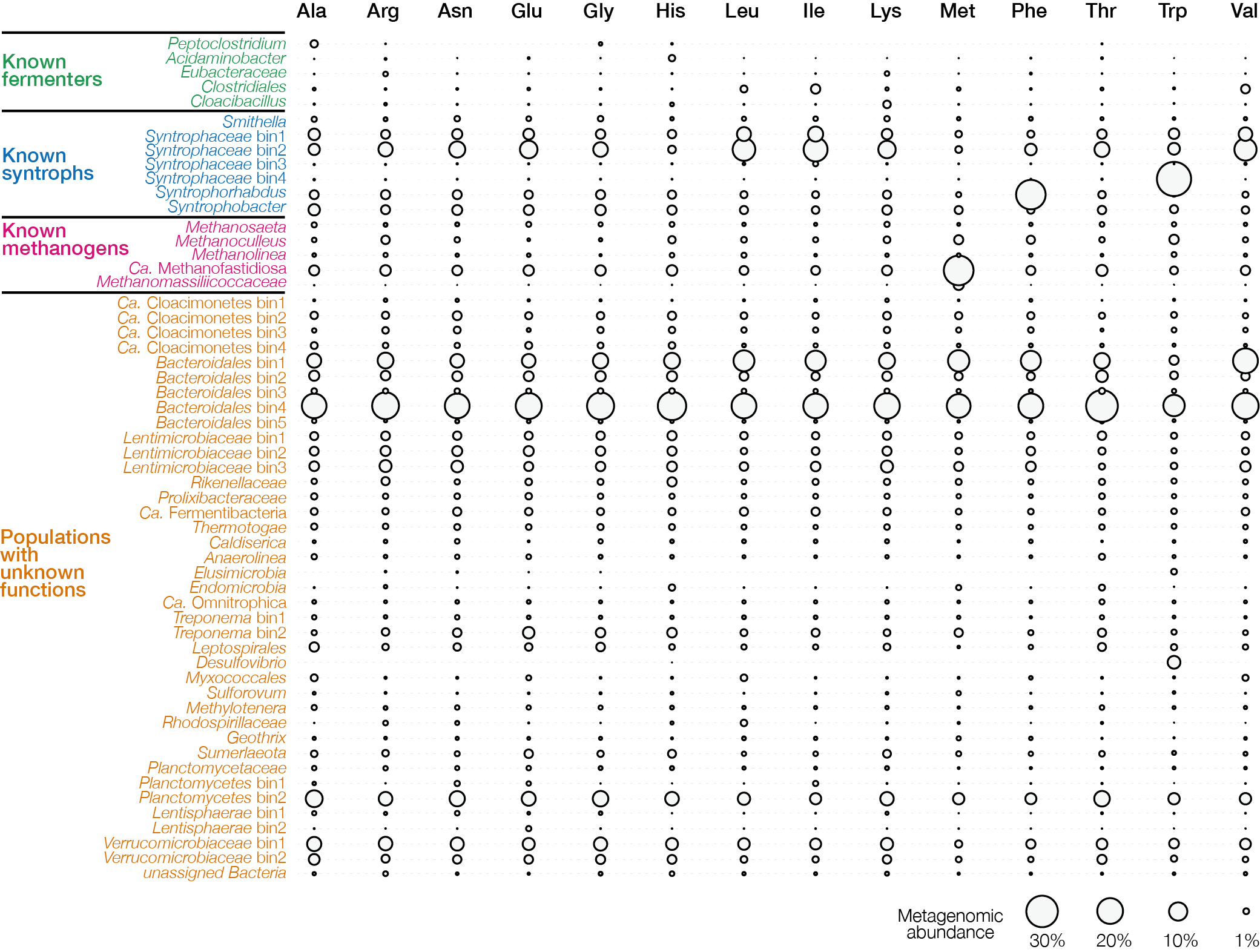


**Supplementary Figure 3.** Metagenomic abundance of bins in each sample.

**
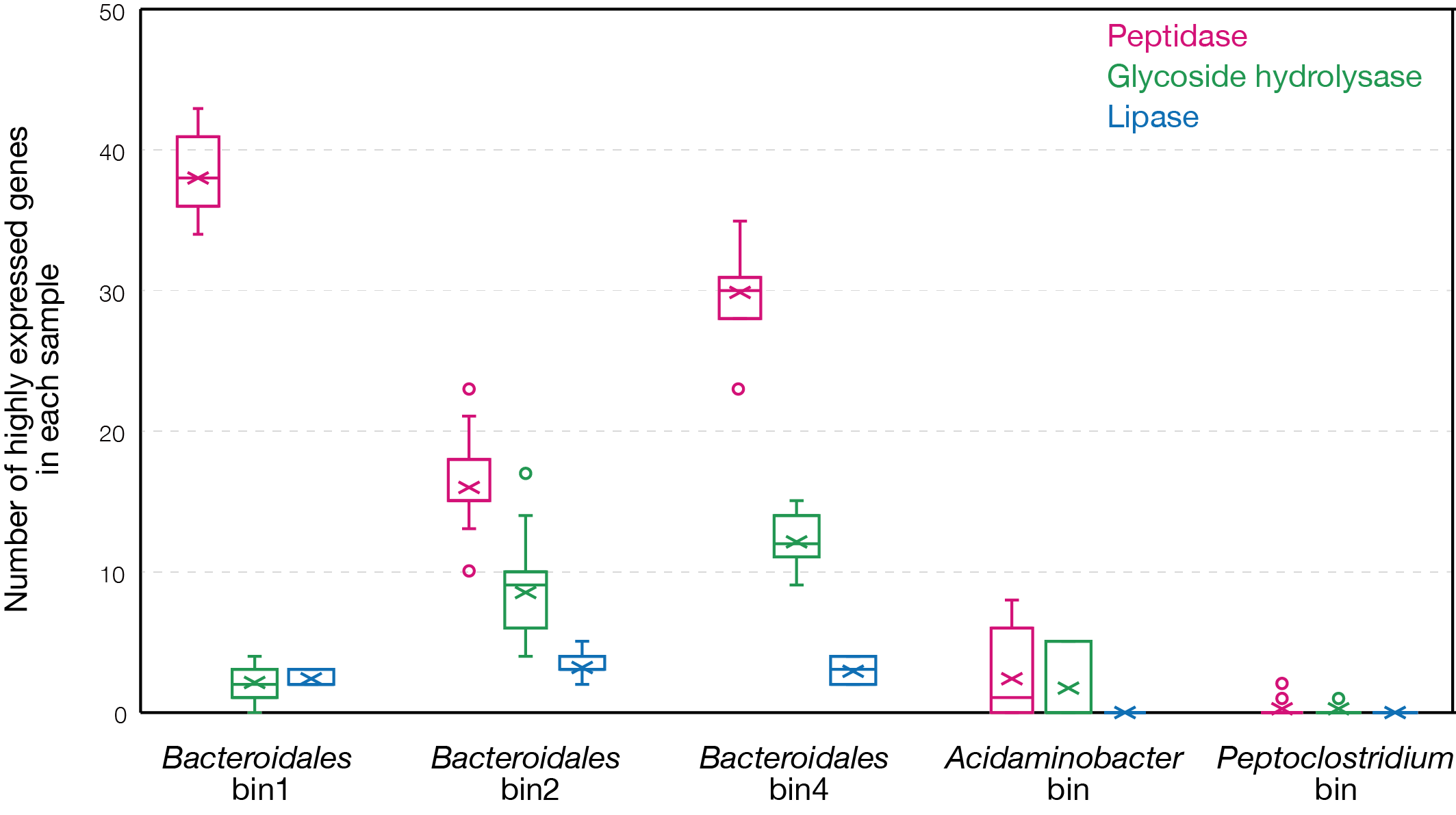
**

**Supplementary Figure 4.** Box-plot of the number of highly expressed secreted polymer hydrolysases in each sample.


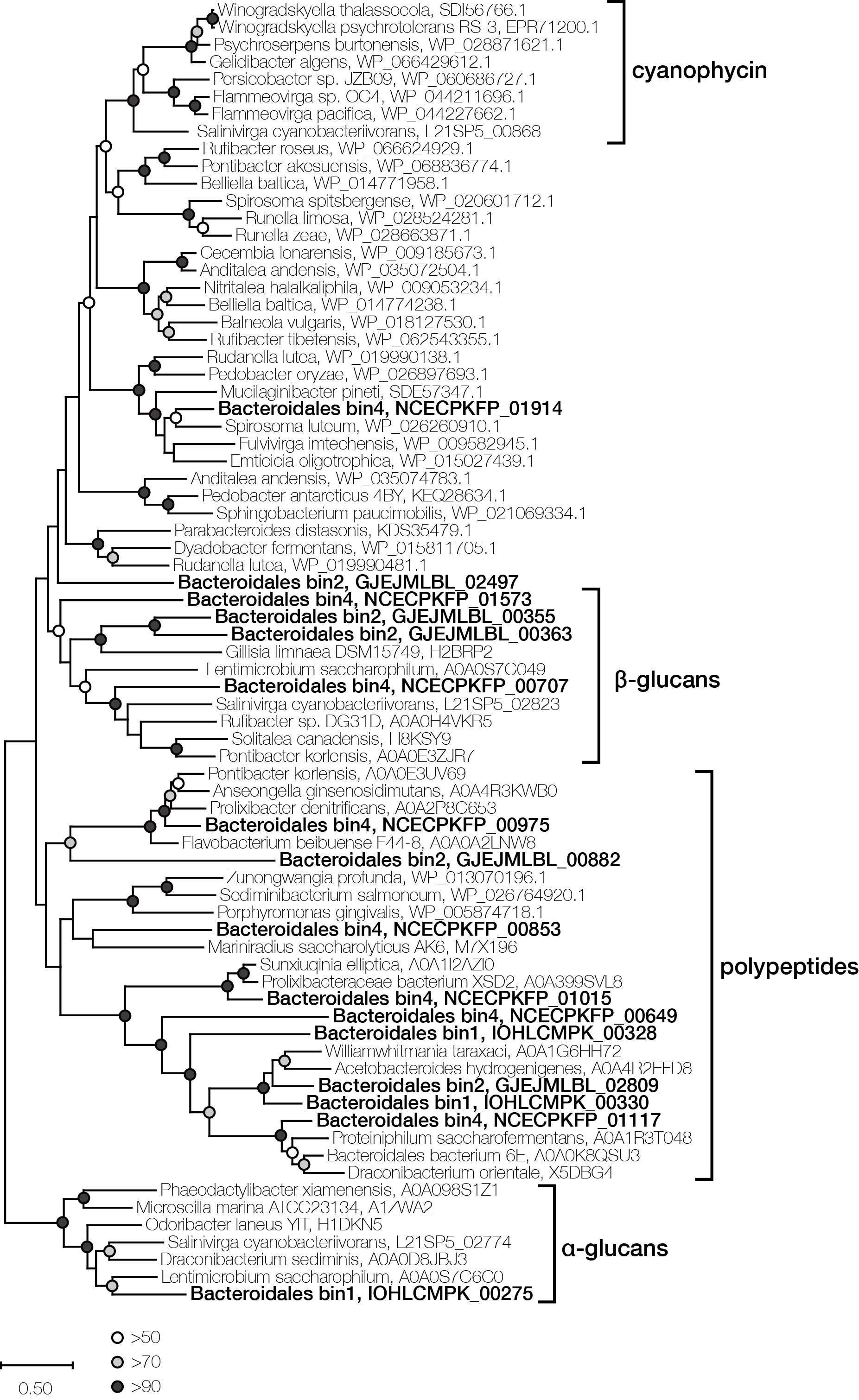


**Supplementary Figure 5.** Maximum likelihood tree of susC-like proteins found in *Bacteroidales* genomes.


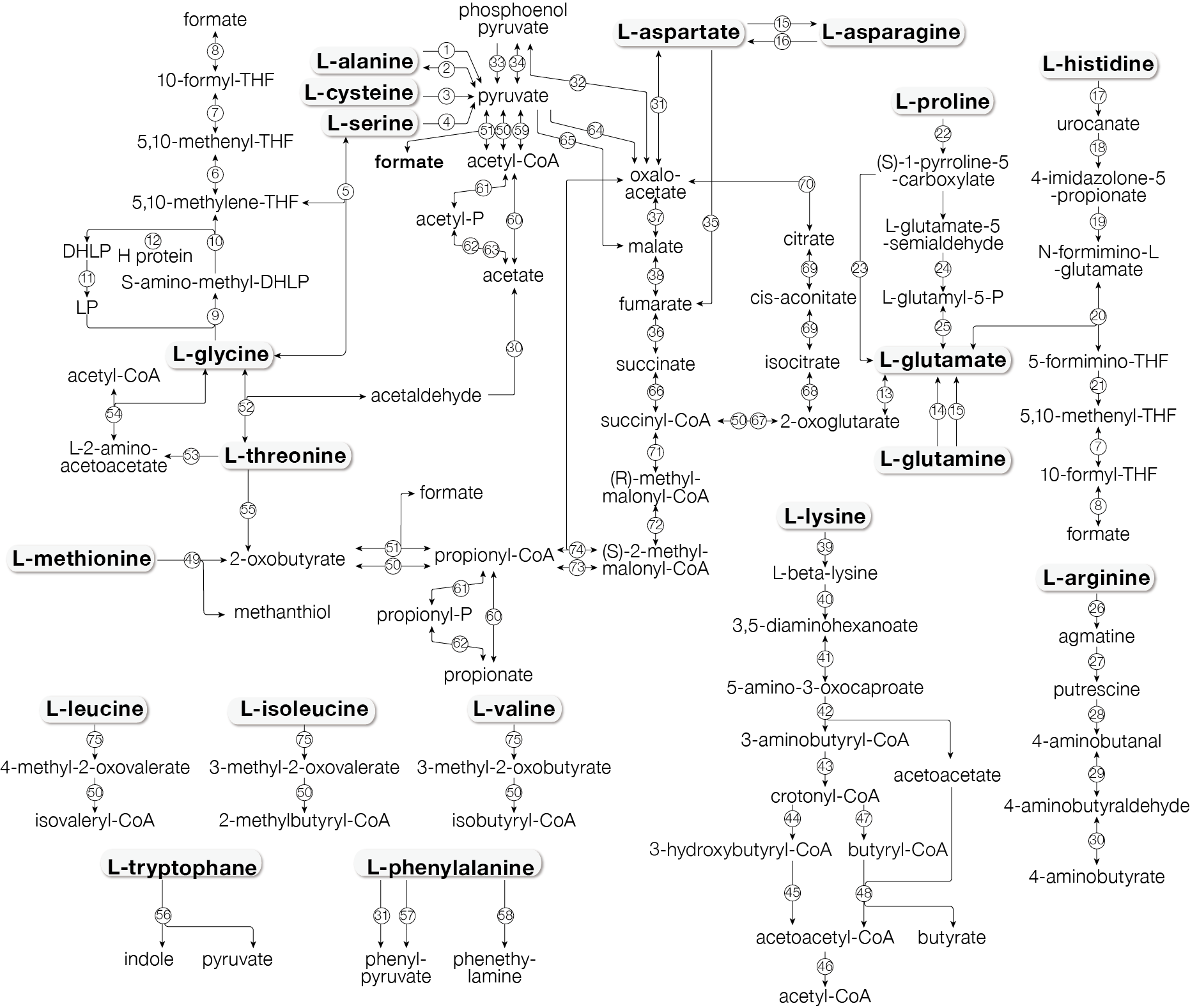


**Supplementary Figure 6.** Amino acids degradation pathways identified in the Bacteroidales bins. Reactions are numbered in the same manner as in Table S5.

## Supplementary Tables

**Supplementary Table 1.** Cultures selected for sequencing and the sequencing project ID on JGI Genome Portal

**Supplementary Table 2.** Families with representative strains in the order Bacteroidales

**Supplementary Table 3.** Predicted number of genes with a potential role in adhesion

**Supplementary Table 4.** Predicted number of genes coding peptidase, glycoside hydrolysase (GH), and lipase

**Supplementary Table 5.** Annotation of Bacteroidales bins

**Supplementary Table 5.** cont

**Supplementary Table 5.** cont

**Supplementary Table 6.** Summary of amino acids degradation capacity


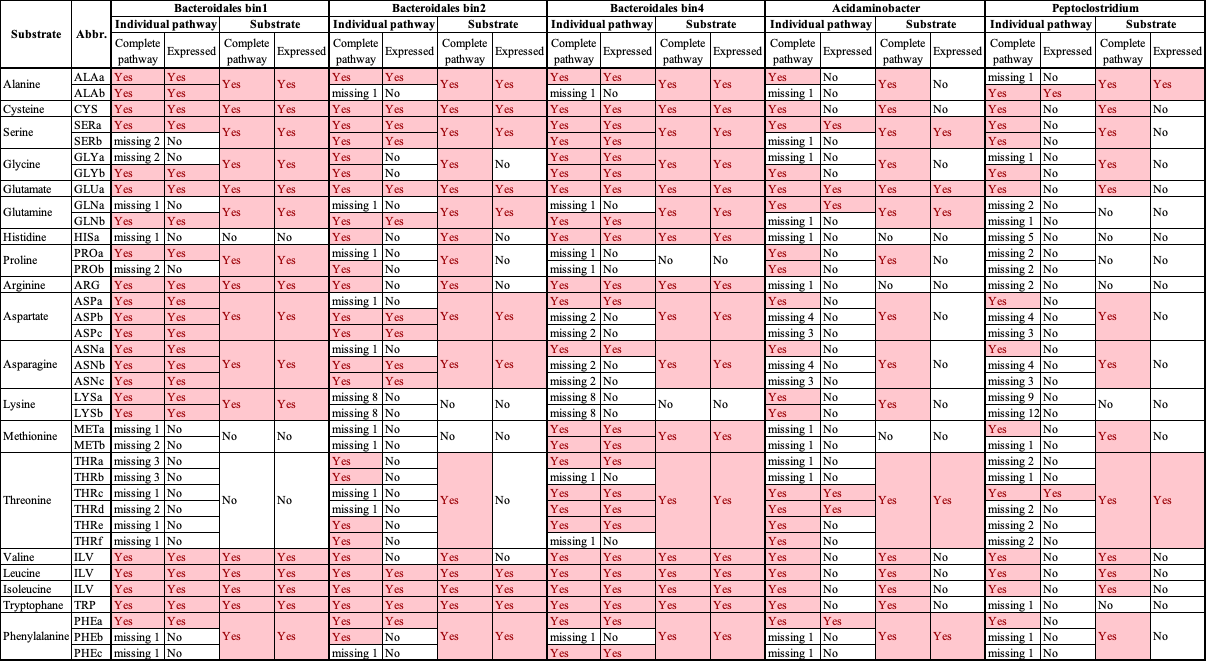


**Supplementary Table 7.** Gene expression related to electron transfer
